# A case report of human infection with avian influenza H10N3 with a complex respiratory disease history

**DOI:** 10.1186/s12879-024-09830-y

**Published:** 2024-09-04

**Authors:** Zhenxi Zhao, Siyi Luo, Yudong Gao, Min Dai, Jun Yan, Ying Yang, Hongwei Li, Yan Zhang, Zhipeng Mao

**Affiliations:** 1 Department of Acute Infectious Disease Prevention and Control (Emergency Response Office), Kunming Center for Disease Control and Prevention, Kunming, Yunnan Province China; 2 Department of Microbiological experiment, Kunming Center for Disease Control and Prevention, Kunming, Yunnan Province China; 3 Department of Acute Infectious Disease Prevention and Control, Guandu District for Disease Control and Prevention, Kunming, Yunnan Province China; 4 Third People’s Hospital of Kunming, Kunming, Yunnan Province China

**Keywords:** Avian Influenza H10N3, Post-COVID19 Pandemic, Case Report, Epidemiological investigations

## Abstract

**Background:**

On March 16th 2024, the first case of Human infection with avian influenza H10N3 since the end of the global COVID-19 Pandemic was reported in Kunming, China. To enhance comprehension of the source of infection and risk factors of the H10N3 virus infection, this case report summarizes the clinical features, epidemiological investigation, and laboratory test results. Provides recommendations for the prevention and control of Human infection with avian influenza H10N3.

**Case presentation:**

A 51-year-old male with a history of COVID-19 infection and a smoking habit of 30 years, worked in livestock breeding and was exposed to sick and dead poultry before falling ill with fever and chills on 28th February 2024. A week later, he was diagnosed with severe pneumonia, influenza, and respiratory failure by the Third People's Hospital of Kunming(KM-TPH). He was discharged on 17th April and none of his 6 close contacts showed any symptoms of illness. Environmental samples taken from the epidemic spot revealed that peacock feces tested positive for avian influenza sub-type H9 and waterfowl specimens showed positive results for avian influenza sub-type H5. Gene sequencing conducted on positive specimens from the patient's respiratory tract by the Chinese Centre for Disease Control and Prevention (CCDC) showed a high degree of similarity (98.6–99.5%) with the strain responsible for the second global case of human infected with H10N3 (reported from Zhejiang, China 2022).

**Conclusions:**

According to the available epidemiological information, there is limited evidence to suggest that H10N3 viruses are excessively lethal. However, adaptive site mutations have been observed in the H10N3 isoform of mammals. While it is unlikely that the H10N3 virus will spread among humans, the possibility of additional cases cannot be entirely ruled out. Symptoms of human infection with H10N3 avian influenza are similar to those of common respiratory infections, which may result in them being overlooked during initial clinical consultations. Therefore, it is essential to improve surveillance of the H10 sub-type of avian influenza and to increase the awareness of hospital-related workers of cases of pneumonia of unknown origin.

**Supplementary Information:**

The online version contains supplementary material available at 10.1186/s12879-024-09830-y.

## Introduction

Avian influenza virus sub-type H10 was first isolated from poultry in 1949 (A/Chicken/Germany/N/1949 [H10N7]) [1].The first human case of avian influenza H10N3 was reported in Jiangsu Province, China in April 2021 [2, 3]. As of the latest WHO weekly report on avian influenza surveillance until March 8th 2024, only two human cases of avian influenza sub-type H10N3 have been reported globally, both in China [4]. This has become a public health concern worldwide [5–7]. In March 2024, a third case of avian influenza H10N3 was reported in Kunming, Yunnan Province, China. This was the first case of avian influenza H10N3 in a patient with a history of COVID-19 infection. This report provides a comprehensive description of the clinical presentation, epidemiological investigation, and laboratory testing to improve our interpretation of the pathogenesis, high-risk exposures, and control policy recommendations associated with human-infected H10N3 virus.

## Case presentation

The case under consideration in this report is a 51-year-old male (BMI 25.35) working in commercial services and livestock breeding in the Kunming, Yunnan province, China. He had a history of COVID19 infection (self-test for SARS-CoV-2 antigen), and he was a smoker with 30-year history. There was no previous history of infectious or chronic diseases, food or drug allergies or blood transfusions. He had a history of exposure to sick and dead poultry within prior to the onset of illness.

### Clinical history

**Fig. 1:**
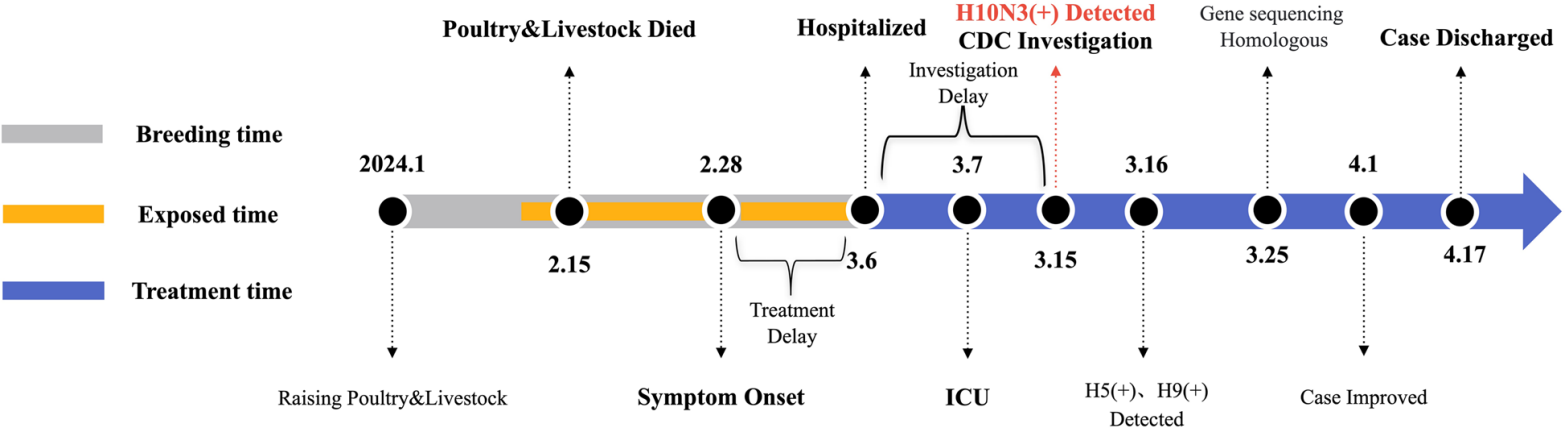
Timeline of the patient's progression for breeding animal, exposure and treatment history

**Fig. 2:**
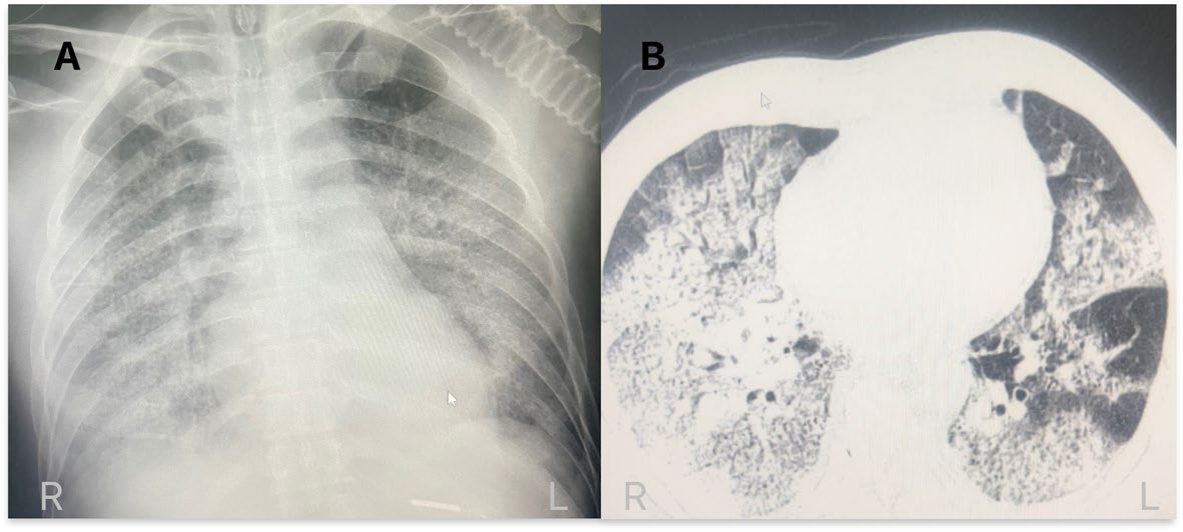
Chest CT in this case of a 51-year-old man with H10N3: Multiple patches and flaky shadows in both lungs, slight thickening of the pleura bilaterally, and a small amount of pleural effusion

On 28th February 2024, the patient experienced clinical symptoms of fever and chills.he self-medicated with ibuprofen, the symptoms did not subside (Fig. 1). From 1st March to 5th March, the patient's condition deteriorated, exhibiting symptoms of influenza-like illness (recurrent high fever, maximum temperature of 39 °C, chills, cough, and shortness of breath). The patient received infusion treatment multiple times at the clinic and the Community Health Service Centre (CHSC) near his residence (the detail was unknown), but his condition did not improve. On 5th March, the patient's chest X-ray at the CHSC revealed an infection in the lower lobe of the right lung. The patient was diagnosed at the Respiratory Department of the KM-TPH on 6 March with (1) severe pneumonia; (2) type I respiratory failure; (3) influenza; (4) hepatic insufficiency; (5) hypercoagulable state; and (6) renal insufficiency(Additional file 1: Table 1). The chest CT scan showed the presence of an infection(Fig. 2). On 7th March, the patient was transferred to the intensive care unit (ICU) due to significant wheezing(PCO_2_ 32 mmHg, PO_2_ 34 mmHg, and SPO_2_ 66%). Between 7 and 15th March, the ICU provided non-invasive ventilator to improve his respiratory functioning(mode S/T, f18 beats/min, IPAP 14 cmH20, EPAP 10 cmH20, Fi02 95%)while administering drugs to control the infection (Omadacycline, Mefenamic acid, Marbofloxacin, and Oseltamivir). On 17th March, the patient's oxygen saturation dropped to 65–75% due to non-cooperation with the non-invasive ventilator. ICU performed orotracheal intubation to improve his respiratory function.

The patient was discharged on 17 April at and was recommended to receive continuous home oxygen therapy and regular follow-up after discharge.

Additional file 1: Table 1 Clinical characteristics of the H10N3 patient(see supplement, P 26).

### Field epidemiology investigation

#### History of suspected exposure and contact

The patient's history of suspected exposure and contact was investigated retrospectively. It was found that the patient followed a consistent and uniform weekday routine without any recent travel abroad. Additionally, the patient had not come into contact with anyone who had respiratory symptoms before experiencing symptoms. Since January 2023, the patient has been farming livestock including chickens, ducks, geese, peacocks, and goats within a particular area. All of the poultry and livestock that the patient raises are purchased from local markets.

The patient is primarily responsible for the upkeep of the farm as well as any sales of animals, he visits once a day to feed the animals. From mid-February to 29^th^ February, there were reported illnesses and deaths among the farmed animals, with approximately 10 chickens, 5 geese, and 5 sheep dying(Table 1). During the period, patients were fed animals and disposed livestock faeces, as well as dead poultry on the farm, without the use of any personal protective equipment (PPE). His family members later stated that the dead poultry and animals had been disposed of by the patient. Though no carcasses were discovered during our epidemiological investigation.

**Table 1:**
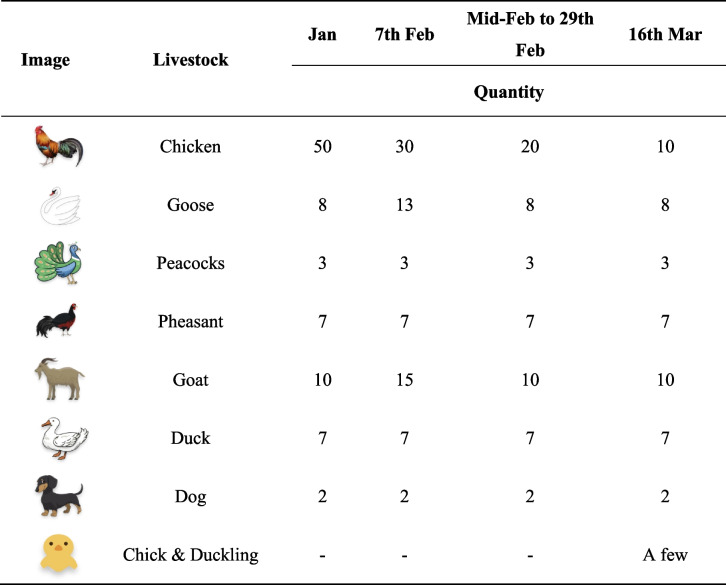
Changes in the type and number of animals kept by patients

#### Epidemic spot investigation

**Fig. 3:**
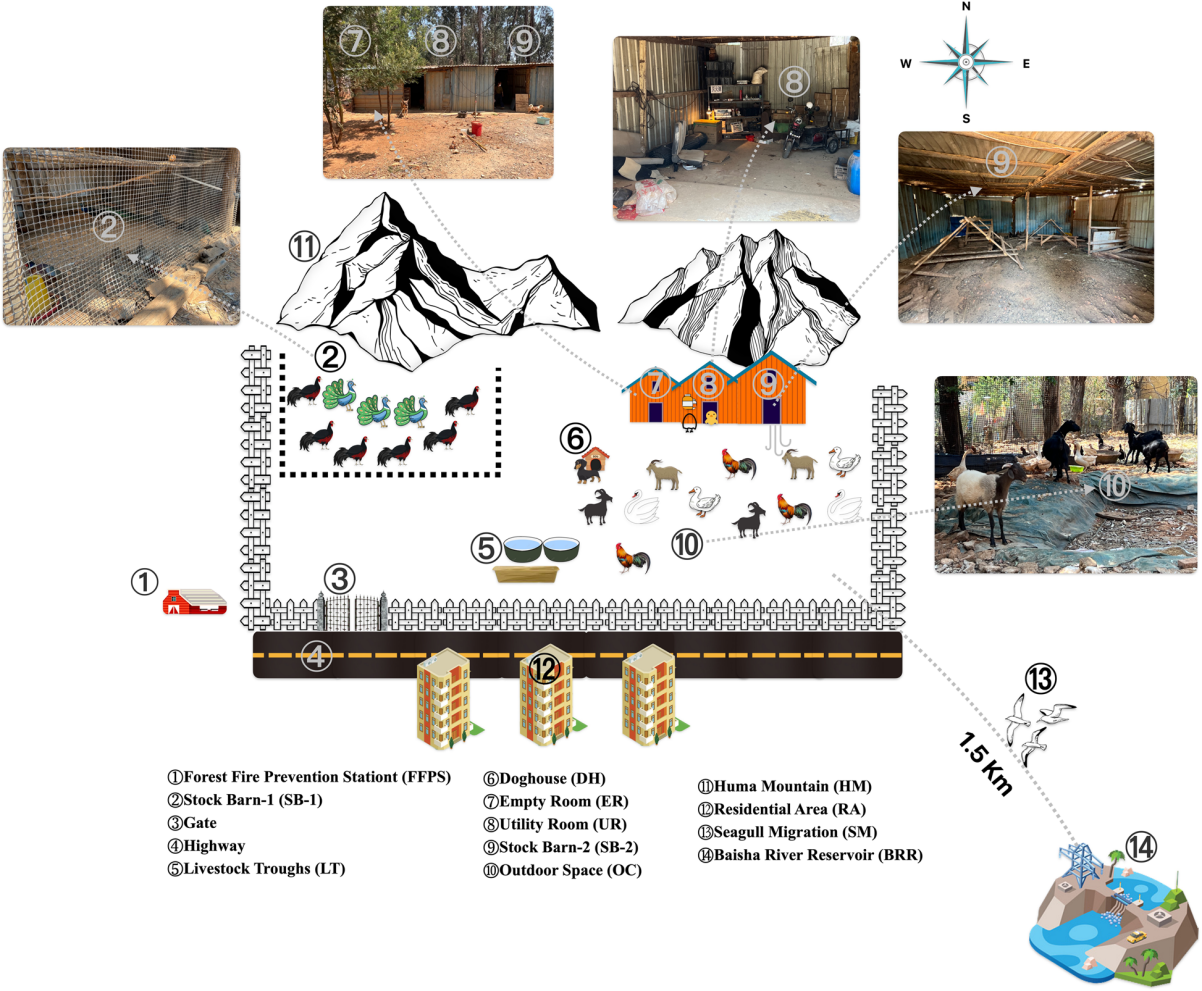
Epidemic spot investigation diagram:UR is used for egg incubation, young bird rearing, feed processing, and Pharmaceutical Storage and Configure. SB-2 serves as a night time penning area for livestock and poultry

The epidemic spot is a wasteland located southwest of the Panlong District of Kunming (KM-PL). The site is located next to the local forest fire prevention station (FFPS) to the west (Fig. 2). To the south, about 10 m away, there is a residential area (RA). The East Baisha River Reservoir (BRR) in Kunming is located approximately 1.5 km southeast. This area is around 1,000 square meters and is open-air. The hygiene conditions in the area are inadequate, with livestock excreta contaminating livestock troughs and personnel areas. The farming pattern in the epidemic spot was mixed captive and free-range farming with no clear regional division. This shows the phenomenon of co-habitation and co-existence of humans, animals, and poultry (Fig. 3).

#### Identification and Tracing of close contacts

According to the Technical Guidelines for the Prevention and Control of Influenza of Human Infection of Animal Origin, person who had direct contact with a patient between February 28th and March 5th 2024 without using any PPE is considered a close contact. In this case, there were a total of 6 close contacts identified for daily monitoring, including 4 family members (3 of whom lived with the patient and 1 who accompanied the patient to the clinic) and 2 management farm workers employed by the patient. None of these individuals had shown any symptoms related to H10N3 or any other respiratory illness within the 10 days following their last exposure to the patient.

### Laboratory testing

On 15th March, TPH submitted a sputum specimen from the patient to KMCDC for testing. The results showed that the patient was positive for influenza A virus sub-type nucleic acid and sub-typing identified influenza virus A (H10N3) sub-type nucleic acid. On the same day, the Yunnan Provincial Disease Control (YNCDC) reviewed the specimen and confirmed the test results. The CCDC gene sequencing results from March 25th indicate that the specimen from this case has a high sequence homology of 98.6–99.5% with the human-infected avian influenza H10N3 sub-type virus that was reported in Zhejiang Province in 2022. The virus may have dual receptor binding since the amino acids 226–228 at the HA (Hemagglutinin) receptor binding site are L/RSG. The amino acid 701 of the PB-2 protein is N, indicating a mutation that is adaptive to mammals. No resistance mutations associated with neuraminidase inhibitor analogues and polymerase inhibitor analogues were detected in the genetic sequences obtained from the patients. Suggests sensitivity to neuraminidase inhibitors (e.g. Oseltamivir) and polymerase inhibitors (e.g. Baloxavir).

The six internal genes identified in the specimens originated from the H9N2 virus.

Nucleic acid tests were conducted twice by the KMCDC on samples from the six close contacts. No influenza A virus was identified. GenBank accession numbers of HA and PB-2 are as follows: HA (PP555669), PB-2(PP555666).

On 16th March, the PLCDC collected the samples from breeding sites, including poultry, livestock, and eggs (Table 2). The KMCDC has detected positive nucleic acid for avian influenza virus A (H9 sub-type) in peacock fecal samples. The Animal Disease Prevention and Control (ADPC)collected 23 biological samples from animals at the exposure site. Among these, three waterfowl anal samples tested positive for influenza virus A (H5 sub-type) nucleic acid, while the remaining samples tested negative.

**Table 2:** Results of environmental and animal specimen sampling

Sample Type	Sample Object	Sample Size	A type					
			**A General**	**H5**	**H7**	**H9**	**H10**	**N3**
**Surface**	Water cup, door handle	1	-	-	-	-	-	-
	Car	1	-	-	-	-	-	-
	Livestock thoughts	1	-	-	-	-	-	-
	Egg	1	-	-	-	-	-	-
	Electromobile	1	-	-	-	-	-	-
	Goat's eye	1	-	-	-	-	-	-
	Chicken yard	1	-	-	-	-	-	-
**Throat swab**	Pheasant	1	-	-	-	-	-	-
	Chicken	1	-	-	-	-	-	-
	Goat	1	-	-	-	" + " Ct:34	-	-
	Dog	1	-	-	-	-	-	-
**Faeces**	Pheasant	1	-	-	-	-	-	-
	Chicken	1	-	-	-	-	-	-
	Chick	1	-	-	-	-	-	-
**Anal swab**	waterfowls	3	-	“ + ”	-	-	-	-
	Pheasant	1	-	-	-	-	-	-
	Chicken	1	-	-	-	-	-	-

“-” Negative result

“ + ” Positive result

## Conclusion

Our report meticulously describes the first case of human-infected avian influenza H10N3 virus since the end of the COVID-19 pandemic. Similar to the onset of the first two globally reported cases [5, 8], human infection with the H10N3 virus initially presents with common symptoms of respiratory disease, such as fever, cough, shortness of breath, and fatigue. These symptoms could be easily overlooked, leading to delayed treatment. The three reported cases worldwide were all caused by delayed treatment in the early stages of the disease, resulting in severe pneumonia, systemic infection, and respiratory failure a week later. The initial two reported H10N3 cases(reported in Jiangsu and Zhejiang) were discharged from the hospital after approximately one month of treatment. However, in this case, the patient was hospitalized for over a month before his infection was initially controlled. Although the inflammation in the lungs had subsided, the patient still required ventilator assistance. It is possibly related to the fact that the patient's exposure history of COVID-19 and prolonged smoking increased the rate of respiratory viruses or even aggravated the severity of the influenza host infection [9, 10]. Moreover, the evidence available could not confirm that the H10N3 virus is more pathogenic compared to other highly pathogenic sub-types of avian influenza. Resistance testing suggests that neuraminidase inhibitors (e.g., oseltamivir) and polymerase inhibitors (e.g. baloxavir) may be used to treat H10N3 virus infection.

This case report was preceded by a clear history of exposure to sick and dead poultry, as well as prolonged exposure to mixed farming. The proximity of case-exposed epidemic spot to BRR may increase the risk of cross-infection between different species of poultry and wildfowl. Research by Wang and Zhang et al. showed that inter-population transmission of avian influenza sub-type H10 and other human-infected avian influenza sub-types occurs through the alteration of a certain gene segment after poultry infection by wild birds carrying the virus, particularly wild waterfowl. This alteration increases the affinity of avian influenza viruses to bind to human receptors [11–13]. Dai et al. also found genetic mutations in this case may increasing the likelihood of human infection avian influenza [14]. Furthermore, the H10N3 sub-type avian influenza virus is a recombinant virus with surface genes derived from the H7 and H9 sub-types of viruses circulating in chickens and ducks [12]. This is despite the limitations of the report, as no poultry carcasses were found and no environmental samples positive for the H10 sub-type were identified. Based on the available epidemiological investigations and genetic sequencing results, the mixed farming pattern of the epidemic spot and the detection of avian influenza viruses (sub-types H5 and H9) in different animals suggests that this is an environment of elevated risk of human exposure to avian influenza viruses including H10N3. Currently, none of the close contacts of the three reported human cases of H10N3 infection worldwide have exhibited clinical symptoms related to avian influenza or tested positive for the H10N3 virus. The H10N3 virus has not acquired the ability for sustained human-to-human transmission, therefore the probability of transmission among humans is currently low [7].

This reported case in Kunming is over 2,000 km away from the locations of the first two cases of homozygosity in Jiangsu and Zhejiang. It could be inferred that avian influenza H10 sub-type viruses have been spreading for a longer period within Yunnan Province even in the wider avian population. Further cases of human-infected H10N3 avian influenza may be reported locally or in other regions of the world. Thus, in terms of the current state of global prevalence. Enhanced surveillance of H10 sub-type viruses in avian influenza-prone areas worldwide is necessary. It's particularly important to reviewed test nucleic acid-positive specimens of excluded seasonal influenza viruses (H1N1, H3N2, B Victoria, B Yamagata) to prevent the spread of this virus.

To reduce the risk of human infection with various animal-borne diseases due to lack of formal regulation and health education. We recommend the following based on this report: (1) The departments of agriculture and rural areas should enhance the regulation and approval process for privately contracted land used for breeding farms. Government agencies should thoroughly review and document the use of contracted land under their jurisdiction. (2) ADPC should improve surveillance and risk identification of animal diseases in live poultry trading markets and private breeding sites. In addition, it is highly recommended that health education be provided to high-risk groups. (3) Clinical staff are advised to improve their sensitivity in the diagnosis and recognition of avian influenza. The focus should be on individuals who have a history of respiratory infectious diseases and smoking. Clinical diagnosis needs to be combined with epidemiological information on the case. (4) The diagnosis must be promptly reported to the CDC for investigation and disposition to interrupt the spread of the disease early.

## Supplementary Information

Supplementary Material 1.

## Data Availability

Data is provided within the manuscript or supplementary information files.The sequence data involved in this study have been deposited in the NCBI GenBank database under accession numbers PP555666 and PP555669.

## Abbreviations

KM-TPH: Third People's Hospital of Kunming
KMCDC: Kunming Centre for Disease Control and Prevention
CCDC: Chinese Centre for Disease Control and Prevention
CHSC: Community Health Service Centre
ICU: Intensive Care Unit
PPE: Personal Protective Equipment
KM-Pl: Panlong District of Kunming
FFPS: Forest Fire Prevention Station
RA: Residential Area
BRR: Baisha River Reservoir
PLCDC: Panlong district for Disease Control and Prevention
YNCDC: Yunnan province for Disease Control and Prevention
ADPC: Animal Disease Prevention and Control
COVID-19: Corona Virus Disease 2019
